# Tissue-specific roles of monoacylglycerol (MAG) in metabolic diseases

**DOI:** 10.1097/IN9.0000000000000087

**Published:** 2026-08-04

**Authors:** Min Young Park, Ashley Mooring, Joanne Bruno, José O. Alemán

**Affiliations:** 1 Laboratory of Translational Obesity Research, New York University Grossman School of Medicine, New York, NY, USA; 2 Translational Research Institute, AdventHealth, Orlando, FL, USA; 3 Holman Division of Endocrinology, Diabetes and Metabolism, Department of Medicine, New York University Grossman School of Medicine, New York, NY, USA

**Keywords:** monoacylglycerol, adipose tissue, metabolism, inflammation, endocannabinoid, cannabinoid receptor

## Abstract

Monoacylglycerol (MAG) is an important bioactive lipid metabolite/intermediate, playing crucial roles in energy homeostasis and cellular signaling. This review explores MAG’s chemical structure, classification, and metabolic pathways. We also discuss the emerging evidence for MAG as a signaling molecule, with particular emphasis on their interactions with cannabinoid receptor 1 and cannabinoid receptor 2 and G-protein-coupled receptor 119. We focus on examining the diverse and tissue-specific functions of MAG in the context of metabolic diseases, including the roles of MAG in nutrient absorption, appetite regulation, obesity, glucose homeostasis, and lipid metabolism. Furthermore, we address therapeutic approaches in modulating MAG metabolism and challenges in development, considering the complexity of targeting peripheral tissues. This review provides insights into the multifaceted roles of MAG in metabolic health and disease, paving the way for novel therapeutic strategies in the management of metabolic disorders.

## 1. Introduction

Lipid metabolism is essential to cell survival as it provides an energy source, structural component, and signaling molecules. A class of lipids called glycerolipids is characterized by a glycerol backbone with long-chain hydrocarbons via carboxylic acid ester linkages ^[1]^. Monoacylglycerol (MAG), one of the glycerolipids, is composed of one fatty acid attached to glycerol. MAG mainly contributes to lipid homeostasis as a precursor in triacylglycerol (TAG) synthesis and an intermediate metabolite during lipolysis, in which its end products, glycerol and fatty acids, are released for energy expenditure. Dysregulated MAG metabolism contributes to hyperlipidemia, hepatic steatosis, and insulin resistance. For example, monoacylglycerol acyltransferase 2 (MGAT2) overactivity in the liver and intestine promotes TAG accumulation and metabolic dysfunction ^[2]^. Moreover, monoacylglycerol lipase (MAGL) hydrolyzes MAG into fatty acid and glycerol, playing a crucial role in TAG hydrolysis and endocannabinoid signaling, with implications for liver diseases and fatty acid metabolism ^[3]^.

In addition to being a substrate and intermediary in lipid metabolism, MAGs are emerging as important signaling molecules. In central and peripheral tissues, they influence insulin secretion, adipose browning, and neurotransmission ^[4]^. Also, MAG signaling modulates macrophage phenotype in adipose tissue, influencing chronic inflammation ^[5]^. The most extensively studied MAG is 2-arachidonoylglycerol (2-AG), which is also known as an endocannabinoid. It acts through binding to its receptor, cannabinoid receptor 1 (CB1R) and cannabinoid receptor 2 (CB2R) ^[6]^. In the forebrain, signaling through CB1R regulates energy homeostasis and appetite. MAG also exhibits cell/tissue-specific roles via CB2R signaling ^[7,8]^. These findings emphasize the impact and diverse roles of MAGs in metabolic regulation and their potential as therapeutic targets.

This mini-review paper covers how MAGs impact metabolic regulation, including nutrient absorption, appetite control, energy homeostasis (diabetes and obesity), cardiovascular function, and immunity. At the end, we address current and potential uses of MAG for therapeutic approaches, as MGAT inhibitors are being explored to suppress TAG synthesis and treat obesity-related liver disease, and inhibitors for MAGL or its isoforms may improve insulin sensitivity and reduce inflammation by preserving signaling MAG species.

## 2. MAG and its metabolism

### 2.1 Description of MAG chemical structure and classification

MAG is a glycerolipid that consists of a glycerol backbone esterified with a fatty acid moiety. Depending on the position of the fatty acid on the glycerol, MAGs can be classified as either 1-MAG or 2-MAG, with the fatty acid attached to the first or second carbon, respectively. This structure provides MAGs with amphiphilic properties, but their solubility relies on the carbon number and saturation degree of the attached fatty acid ^[9]^. As expected, MAGs with longer chain lengths (≥10 carbon atoms) have low solubility in buffer alone but increased solubility in bile salt solutions. For example, 1-oleoylglycerol and 2-oleoylglycerol (2-OG) are solubilized identically by bile-salt solutions, but 2-palmitoylglycerol (2-PG) has a higher solubility than 1-palmitoylglycerol due to the lower melting point of 2-PG. Nevertheless, their amphipathic nature makes MAGs dynamic, important intermediates in lipid metabolism and functional in both the aqueous environment of cells and lipid membranes.

### 2.2 Endogenous MAG synthesis and degradation

Endogenous MAGs are short-lived, intermediary lipids that derive mostly from the degradation of phospholipid and neutral lipids ^[10]^. Being the most extensively studied MAG, 2-AG primarily originates from membrane phospholipids through the sequential action of phospholipase C (PLC) and diacylglycerol lipase (DAGL). Upon activation of G protein-coupled receptors (GPCRs), PLC converts phosphatidyl bisphosphate (PIP_2_) to inositol trisphosphate (IP_3_) and diacylglycerol (DAG). DAG is then hydrolyzed by DAGL, producing 2-MAG (eg, 2-AG) ^[11]^. Alternative pathways of 2-AG synthesis derive from the extracellular cleavage of 2-arachidonoyl-lysophospholipids by glycerophosphodiesterase 3, lipid phosphate phosphatases, and two members of ecto-nucleotide pyrophosphatase/phosphodiesterases (ENPP6-7) ^[12]^. 2-AG can also be synthesized by dephosphorylation of arachidonoyl-lysophosphatidic acid (LPA) or by the sequential action of PLA1 and lysophospholipase C (lyso-PLC) ^[13,14]^. It is also worth noting that some factors affect the biosynthesis of MAGs, such as oxyradical stress increases the biosynthesis of 2-AG via involvement of nicotinamide adenine dinucleotide phosphate (NADPH) oxidase ^[12]^.

**Figure 1.**
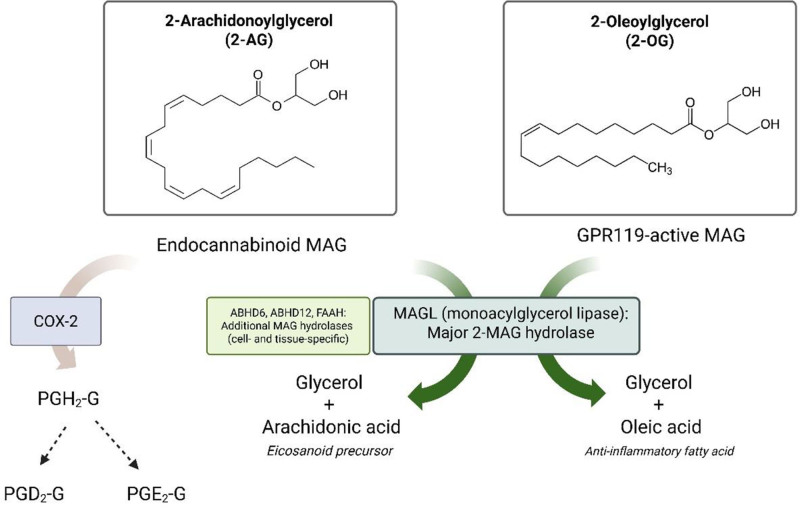
**Metabolism of two well-characterized signaling monoacylglycerols (MAGs).** Monoacylglycerol lipase (MAGL) is the major enzyme for 2-MAG hydrolysis, and it hydrolyzes 2-AG and 2-OG into glycerol and fatty acids, arachidonic acid and oleic acid, respectively. Besides MAGL, α/β-hydrolase domain-containing 6 (ABHD6) and 12 (ABHD12), and to a lesser extent fatty acid amide hydrolase (FAAH), contribute to 2-AG hydrolysis and shape distinct pools of 2-AG in a cell- and tissue-specific manner ^[10,15–17]^. Once hydrolyzed, arachidonic acid can be further oxidized by cyclooxygenase-2 (COX-2) to produce prostaglandins, whereas oleic acid increases fatty acid oxidation via NAD-dependent deacetylase sirtuin-1 (SIRT1)-peroxisome proliferator-activated receptor gamma coactivator 1-α (PGC-1α)-dependent modulation of PPAR signaling ^[18,19]^. In addition to hydrolytic catabolism, 2-AG can be oxidized into PGH_2_-G, which is converted by PGD synthase to PGD_2_-G (DP1 and PPARγ receptor activation via 15d-PGJ_2_-G) or by PGE synthase to PGE_2_-G ^[20–23]^. 2-AG, 2-arachidonoylglycerol; 2-OG, 2-oleoylglycerol; ABHD12, α/β-hydrolase domain containing 12 COX-2, cyclooxygenase-2; ABHD6, α/β-hydrolase domain containing 6; FAAH, fatty acid amide hydrolase; MAG, monoacylglycerol; MAGL, monoacylglycerol lipase; PPAR, peroxisome proliferator-activated receptor.

Once synthesized, MAG is degraded by MAGL, producing glycerol and fatty acid (Figure 1). In addition to MAGL being a primary enzyme (~85%), 2-MAG can be also hydrolyzed by fatty acid amide hydrolase, α/β-serine hydrolase domain 6 (ABHD6) (4%), and α/β-serine hydrolase domain 12 (9%) in mouse brain ^[24]^. Subcellular localization and/or cell/tissue-specific expression might also contribute to 2-AG degradation. For example, ABHD6 is primarily expressed in GABAergic neurons and astrocytes located postsynaptically ^[15]^. Also, expression levels of ABHD6 are relatively higher compared to MAGL in J774 macrophages ^[20]^.

Among MAGs, 2-AG has been studied extensively in the brain as it acts as a signaling molecule in the central nervous system. The synthesis and metabolism of 2-AG in the brain are highly cell type-specific. In neurons and astrocytes, DAGL α is the primary enzyme responsible for 2-AG synthesis, activated downstream of G protein-coupled and glutamate receptor stimulation ^[25–27]^. DAGL β predominantly mediates 2-AG synthesis in microglia, representing a distinct biosynthetic pathway in the brain’s resident immune cells ^[26]^. Regarding 2-AG degradation, at least eight different enzymes participate ^[28]^. MAGL is the major hydrolytic enzyme in neurons and astrocytes; in astrocytes specifically, MAGL-mediated conversion of 2-AG to arachidonic acid is coupled to downstream cyclooxygenase-2 (COX-2) activity and constitutes an important source of neuroinflammatory prostaglandins ^[26,29]^. In microglia, α/β-hydrolase domain-containing protein 12 (ABHD12) serves as the principal hydrolase for secreted extracellular 2-AG, modulating cannabinoid receptor activation and microglial immune homeostasis ^[26]^. This cell type-specific partitioning of 2-AG biosynthesis and catabolism has important implications for the interpretation of tissue-level endocannabinoid measurements and the design of cell-selective therapeutic strategies. These highlight that multiple enzymes are intricately orchestrated and regulate MAG homeostasis and suggest MAG hydrolysis as a therapeutic target.

### 2.3 Exogenous sources of MAGs

MAG also derives from the absorption of TAG in various dietary sources, such as butter and oils. Dietary TAGs are hydrolyzed into DAG, and subsequently to MAGs by pancreatic lipase ^[30]^. In the intestinal lumen, MAGs are absorbed at the brush border of enterocytes primarily via passive diffusion ^[31]^. MAG uptake can also be facilitated via specific transport proteins in human intestinal Caco-2 cells ^[32]^. In this study, the authors demonstrate that trypsin preincubation decreased sn-2-monoolein uptake, and competition studies with oleic acid inhibited sn-2-monoolein uptake. This suggests a common uptake pathway for MAG and fatty acids, and a plasma membrane fatty acid binding protein as a putative carrier. Moreover, Lagakos et al have shown that the rate of MAG transfer is 7-fold faster from liver fatty-acid binding protein (FABP) to phospholipid membranes than from membranes to membranes by using transfer of a fluorescent MAG analog ^[33]^. Although equilibrium binding affinities of MAG were ∼2-fold lower when compared to fatty acids, these results provide strong support for MAG as a physiological ligand for liver FABP and further suggest that liver FABP functions in the transport of MAG, as well as hepatic degradation of 2-MAG ^[34]^.

Once entering enterocytes, MAGs are re-esterified into TAGs in the endoplasmic reticulum. Along with cholesterol and phospholipids, newly formed TAGs are packaged into chylomicrons. Circulating chylomicrons then transport lipids into various tissues, including adipose tissue for energy storage and muscle for energy utilization, respectively.

Moreover, intracellular MAG can be derived from the hydrolysis of TAG. TAG is hydrolyzed by adipose triglyceride lipase (ATGL), generating DAG. Subsequently, hormone-sensitive lipase (DAGL/HSL) catalyzes the breakdown of DAG into MAG and fatty acid. Regulation of MAG synthesis is influenced by various factors, including fatty acid availability, hormonal signals, and lipase activity ^[35–38]^.

## 3. Role of MAGs in metabolism

MAGs can act both as substrate/intermediate to regulate anabolic and catabolic pathways in lipid metabolism. MAG could be converted to DAG by MGAT and subsequently to TAG by diacylglycerol acyltransferases (DGAT) for excess energy storage. On the other hand, MAGs can serve as precursors for key fatty acids, such as arachidonic acid, oleic acid, or palmitic acid, which can be utilized to generate ATP. MAGL is the major enzyme catalyzing the hydrolysis of MAGs into glycerol and fatty acids (Figure 1) ^[10]^. MAG structure and its concentrations may also impact its hydrolysis, leading to different enzyme affinity. MAGL prefers 2-MAGs; therefore, 2-MAGs exhibit more bioactive compared to 1-MAG, while ABHD6, an isoform of MAGL, has a stronger preference for relatively more hydrophilic 1-AG than 2-AG ^[15,39,40]^. Also, cell-specific expression and activities of MAGL and its isoforms (ABHD6 and ABHD12) are major in vivo determinants of 2-AG and arachidonic acid levels in the brain, spleen, lung, and liver ^[26,29,41]^. Beyond hydrolytic metabolism, 2-AG can also serve as a direct substrate for COX-2, which oxygenates 2-AG to prostaglandin glycerol ester (PG-G) and, to a lesser extent, hydroxyeicosatetraenoic acids ^[21]^. PG-G is further metabolized into other prostaglandins (Section “MAG and inflammation/immunity”). These suggest that 2-AG is subject to context-dependent metabolism, where substrate availability, allosteric competition, and inflammatory conditions can influence its conversion into bioactive prostaglandins, and COX-2-mediated oxygenation may modulate 2-AG signaling for its action as an endocannabinoid ^[42–44]^. Moreover, 2-OG hydrolysis liberates oleic acid, which contributes to promoting anti-inflammation ^[45]^. Also, phosphorylation of 2-AG by acyl glycerol kinase(s) creates LPA ^[46]^, which can activate different cellular responses including cell proliferation, differentiation, and inflammation via the GPCR-mediated MAPK, PI3K/Akt, or JNK/P38 MAPK pathway ^[47]^. Finally, lipoxygenases can oxidize 2-AG, producing hydroperoxy derivatives of 2-AG ^[48]^. Therefore, 2-AG regulation by metabolic enzymes coordinates overall cellular consequences.

### 3.1 Energy metabolism

#### 3.1.1 Contribution of MAGs to energy homeostasis

MAGs play a crucial role in energy storage and metabolism as an intermediate metabolite in the synthesis and breakdown of TAG. For energy storage, the MGAT pathway is the primary route for TAG biosynthesis and dietary fat absorption in the small intestine ^[49]^. MGAT1, which converts MAG to DAG in this pathway, is highly expressed during adipocyte differentiation and may regulate basal adipocyte free fatty acid retention ^[50]^. In the small intestine, MGAT2 (encoded by *Mogat2* in mice and *MOGAT2* in humans) is highly expressed, and global *Mogat2*-deficient mice are protected against diet-induced obesity and glucose intolerance through delayed fat entry into circulation, increased energy expenditure, and elevated postprandial GLP-1 levels ^[51,52]^. Intestinal re-expression of human MGAT2 in *Mogat2*-deficient mice partially restored these phenotypes, confirming MGAT2 as a key determinant of metabolic efficiency ^[53]^. In the catabolic pathway, when MAGs are hydrolyzed into glycerol and fatty acid by MAGL, fatty acid can enter β-oxidation for energy production. Therefore, MAGL engages in energy homeostasis through fat mobilization, endocannabinoid (eg, 2-AG) degradation ^[54]^, and providing precursors (eg, arachidonic acid for prostaglandin synthesis). MAGL overexpression in the mouse small intestine showed reduced MAG levels (eg, 2-AG), increased food intake, fat mass, and body weight, and reduced energy expenditure, which led to obesity. Conversely, global deletion of MAGL in mice leads to increased MAG levels, reduced weight gain, and delayed lipid absorption, resulting in a leaner phenotype and improved metabolic profile ^[55]^. Lastly, MAGL deficiency enhances energy expenditure and protects against obesity even without high-fat feeding ^[56]^.

Overall, both models of MGAT2 and MAGL deficiency promote metabolically lean phenotypes by preserving MAG availability. MGAT2 knockout (KO) primarily impairs intestinal MAG re-esterification into TAG and thereby exerts a direct effect on dietary fat absorption and systemic lipid metabolism, while MAGL KO broadly elevates MAG and alters endocannabinoid signaling in a cell/tissue-specific manner. These findings suggest that enzymes involved in MAG metabolism, such as MGAT and MAGL, could be potential therapeutic targets for treating metabolic disorders associated with elevated TAG levels ^[49]^.

### 3.2 Signaling molecules

#### 3.2.1 MAGs as bioactive lipids and its receptors/binding proteins

In addition to being a precursor or product in lipid metabolism, MAGs play a role as a signaling molecule. Individual MAGs exhibit tissue-specific interactions with receptors/proteins, including cannabinoid receptor, fatty acid binding protein, G-protein-coupled receptor 119 (GPR119), and retinoic acid binding protein, and activating/leading to various signaling pathways.

**Figure 2.**
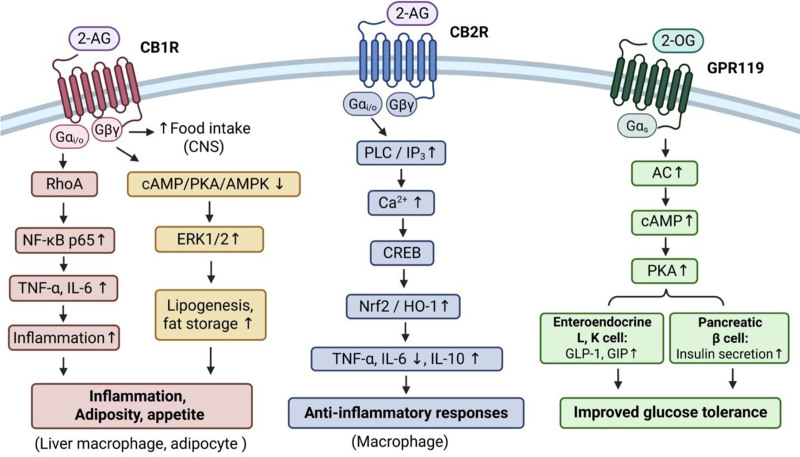
**MAG signaling pathway leading to inflammatory and metabolic responses**. (Left) In Kupffer cells, Gα_i/o_-coupled CB1R induces inflammation via RhoA-NFκB p65 nuclear translocation activation, releasing TNF-α and IL-6 in liver fibrosis ^[60]^. In adipocytes, Gβγ-coupled CB1R reduces cAMP, PKA phosphorylation, and AMPK activation, stimulating ERK1/2, which induces lipogenesis and increases fat storage ^[61–63]^. Additionally, in hypothalamic appetite circuits, 2-AG released postsynaptically from orexigenic AgRP/NPY and lateral hypothalamic orexin/MCH neurons acts as retrograde messengers to activate presynaptic G_i/o_-coupled CB1R on GABAnergic neurons, reducing transmitter release, disinhibiting these hunger-promoting neurons, and ultimately increasing food intake ^[64–66]^. (Center) CB2R predominantly recruits Gα_i/o_ proteins and can activate the PLC/IP_3_/Ca^2+^ pathway, leading to nuclear factor E2-related factor 2 (Nrf2) translocation and heme oxygenase 1 (HO-1) signaling, which reduces TNF-α and IL-6 while induces IL-10 secretion ^[67]^. (Right) GPR119 activation by 2-oleoylglycerol (2-OG) improves glucose-dependent insulin secretion. G_s_-coupled GPR119 increases cAMP levels, which induces GLP-1 and GIP release in gastrointestinal L and K cells, respectively, which stimulate insulin secretion in pancreatic β-cells ^[68–70]^. 2-AG, 2-arachidonoylglycerol; 2-OG, 2-oleoylglycerol; AC, adenylyl cyclase; AgRP, agouti-related peptide; cAMP, ; CB1R, cannabinoid receptor 1; CB2R, cannabinoid receptor 2; CREB, cAMP response element-binding protein; GIP, glucose-dependent insulinotropic polypeptide; GLP-1, glucagon-like peptide 1; GPR119, G protein-coupled receptor 119; IP3, inositol 1,4,5-trisphosphate; MCH, melanin-concentrating hormone; NPY, neuropeptide Y; PKA, protein kinase A; PLC, phospholipase C.

(i) CB1R and CB2R: 2-AG has been the most intensively studied MAG for its role as an endocannabinoid, and it binds both G-protein-coupled receptors, known as CB1R and CB2R, activating pro- and anti-inflammatory signaling pathways. CB1R is predominantly expressed throughout the brain ^[57]^, whereas CB2R has 44% amino acid homology to CB1R and is primarily localized on cells of the immune system ^[58,59]^. CB1R can interact with either Gα_i/o_ or Gβγ subunits. In Kupffer cells, Gα_i/o_-coupled CB1R induces inflammation via RhoA-NFκB p65 nuclear translocation activation, releasing tumor necrosis factor alpha (TNF-α) and interleukin (IL)-6 in liver fibrosis ^[60]^. In adipocytes, Gβγ-coupled CB1R reduces cyclic adenosine monophosphate (cAMP), protein kinase A (PKA) phosphorylation, and adenosine 5′-monophosphate-activated protein kinase (AMPK) activation, stimulating ERK1/2, which induces lipogenesis and increases fat storage ^[61]^. On the other hand, CB2R predominantly recruits Gα_i/o_ proteins and can activate the PLC/IP_3_/Ca^2+^ pathway, leading to nuclear factor E2-related factor 2 (Nrf2) translocation and heme oxygenase 1 (HO-1) signaling, which reduces TNF-α and IL-6 while inducing IL-10 secretion (Figure 2) ^[67]^. Although less described, CB2R can also induce other signaling pathways, including PKA activation via a pro-inflammatory response ^[71]^.

The relevance of CB1R and CB2R with metabolic dysregulation has been demonstrated in the brain, adipose tissue, and adipose tissue macrophages (ATM). CB1R blockade attenuates diet-induced obesity-associated inflammation through alterations in ATM miRNA expression that promote M2 ATM polarization and macrophage egress from adipose tissue ^[72,73]^. Although CB2R is predominantly expressed in the myeloid lineage, its low expression was also detected, reflecting resident macrophages ^[74]^. In addition, administration of the CB2R-selective agonist JWH-133 was found to attenuate inflammation in white adipose tissue of diet-induced obese mice by restraining M1 macrophage polarization via the Nrf2/HO-1 pathway ^[67]^, and beneficial effects on fat mass and adipocyte size were also reported after administration of the less selective CB2R agonist JWH-015 ^[75]^.

(ii) GPR119: GPR119, a GPCR expressed in pancreatic β-cells and enteroendocrine cells, has emerged as a promising target for type 2 diabetes treatment ^[68]^. GPR119 agonists include *N*-oleoylethanolamine ^[76]^, *N*-Oleoyl-dopamine ^[77]^, lysophosphatidylcholine ^[78]^, and 2-OG ^[69]^. Despite their structural similarities between GPR119 and cannabinoid receptors, the endogenous ligands of GPR119 and CB1R selectively recognize their own receptors ^[79]^. Also, GPR119 is highly expressed in pancreatic β cells and intestinal enteroendocrine L cells ^[80]^, while cannabinoid receptors are more widely distributed in various tissues as well as the gastrointestinal tract. Activation of GPR119 by endogenous lipids like MAGs and synthetic agonists increases intracellular cAMP, leading to enhanced glucose-dependent insulin secretion and incretin hormone release ^[81]^. For example, studies have shown that GPR119 agonists improve glucose tolerance in mice through glucagon-like peptide-1 (GLP-1) and cholecystokinin (CCK) release, with effects dependent on receptor activation ^[70]^. Also, Mandoe et al reported that the 2-MAG moiety of dietary fat appears to be responsible for the fat-induced release of GLP-1 in humans ^[82,83]^. The receptor exhibits broad and biased signaling, including G_αs_, G_αq_, and G_αi_ pathways, as well as β-arrestin recruitment ^[70]^. Synthetic GPR119 agonists have demonstrated potential in regulating glucose homeostasis and reducing body weight in rodent models, making them attractive candidates for treating type 2 diabetes and related metabolic disorders ^[81]^. Hansen et al also reported that 2-OG is a GPR119 and signals GLP-1 release in the human intestine ^[69]^.

Other proteins that are demonstrated to interact with MAGs include retinol-binding protein 2 (RBP2), a member of FABP, as shown that intestinal MAG concentration rises in the absence of Rbp2 in intestinal (jejunum) cells. Purification of human RBP2 examined the highest affinity of sn-1 and sn-2 MAGs containing polyunsaturated C18-C20 acyl chains, suggesting that Rbp2 modulates MAG metabolism and its downstream signaling ^[84]^. In addition, Lagakos et al demonstrated that MAG binds to liver FABP (FABP1) in vitro and mouse liver cytosol ^[33]^. As discussed in section 2.3, MAG is shown to bind liver FABP, which is expressed in both liver and intestinal mucosa, and its global deletion altered FA and MAG metabolism. In line with potential aspects of MAGs as a signaling molecule, Shanbhag et al reported that neuronal calcium sensor Hippocalcin is a putative protein ligand for 1-PG via a chemoproteomics approach, suggesting a modulation of calcium sensing and downstream signaling in the mammalian brain.

## 4. MAGs in metabolic disorders

This section covers diverse actions of MAG in different tissues and pathophysiological conditions, and factors regulating 2-MAGs in distinct conditions.

### 4.1 MAGs in intestinal epithelium: dietary fat absorption and incretin regulation

Upon digestion, dietary lipids are predominantly absorbed as MAG and fatty acids from micelles at the apical membrane of the intestinal epithelium via selective diffusion and/or protein-mediated processes, with potential competition for the same transport proteins ^[32]^. Once these micellar components cross the plasma membrane of enterocytes and diffuse into the cytoplasm, they encounter the smooth endoplasmic reticulum, where re-esterification of lipids into TAG takes place. The synthesized TAGs are then packaged into chylomicrons for secretion into lymph ^[85]^.

Studies have shown that the site of MAG and FA entering enterocytes significantly affects its metabolic fate, such as apical delivery favoring TAG synthesis and basolateral delivery promoting phospholipid formation and fatty acid oxidation ^[86,87]^. Both MAG and fatty acids show a 10-fold higher TAG to phospholipid (TAG:PL) ratio when compared between apical vs basolateral delivery in rodent intestines. Apically absorbed dietary lipids are primarily packaged into chylomicrons or stored in cytoplasmic lipid droplets, while basolaterally absorbed lipids may undergo various fates, including oxidation, structural lipid synthesis, or re-secretion ^[88]^, suggesting compartmentation/ polarized lipid metabolism allows enterocytes to integrate and regulate overall lipid flux, in which MAG and FA would affect their uptake and utilization.

Douglass et al described the systemic and direct effect of MAGs on intestinal lipid absorption in a MAGL-deficient mouse model, as an increase in MAG delayed intestinal lipid and shifted systemic metabolic changes towards resistant to diet-induced obesity ^[55]^. MAG also plays a role in hepatic uptake of cholesteryl esters from circulating lipoproteins. Mortimer et al demonstrated that the addition of 1- or 2-monostearoylglycerol in emulsion decreased the amount of cholesteryl ester found in the liver, without affecting TAG ^[89]^. It suggests that saturated MAG produced by the action of lipoprotein lipase may attenuate uptake of TAG-depleted remnant lipoprotein particles in the plasma into the liver.

In parallel, intestinal 2-MAGs modulate incretin secretion via GPR119 activation. Mandoe et al reported that the 2-MAG moiety of dietary fat appears to be responsible for the fat-induced release of GLP-1 in humans ^[83]^. The authors identified 2-OG as a crucial factor for GLP-1 secretion by comparing the effects of tributyrin (short-chain fatty acids), olive oil (long-chain fatty acids and 2-oleoyl glycerol), and 1,3-dioctanoyl-2-oleoyl glycerol (C8-dietary oil, producing medium-chain fatty acids and 2-oleoyl glycerol) on hormone responses in healthy male subjects. Both C8-dietary oil and olive oil, which produce 2-OG during digestion, stimulated GLP-1 release, while tributyrin, which does not produce 2-OG, failed to stimulate GLP-1 release. Moreover, Hansen et al reported that MAGs (2-OG, 2-linoleoylglycerol [2-LG], or 2-PG) can activate GPR119 in Cos-7 cells, and oral administration of 2-OG significantly increased plasma incretin hormone GLP-1 and glucose-dependent insulinotropic polypeptide (GIP) levels in individuals, suggesting that GPR119 is a fat sensor and MAG is a ligand/an agonist for incretin production ^[69]^.

Additionally, Lee et al reported that RBP2 is a protein that interacts with MAGs, which may modulate downstream metabolic effects ^[90]^. RBP2 is a member of the FABP family expressed in the intestine, and the absence of RBP2 in mice elevated intestinal 2-MAG levels (2-AG, 2-LG, and 2-PG), which was associated with increased GIP levels. Notably, elevated circulating MAG levels with systemic accumulation from MAGL deletion delayed lipid absorption and improved energy balance, while localized intestinal MAG elevation in RBP2 deletion enhanced gut hormone signaling and promoted obesity, highlighting MAGs’ context-dependent roles in metabolism.

### 4.2 Role of MAGs in appetite regulation in CNS

Role of MAGs in regulating metabolic pathways is also closely linked with signaling in the brain. Its relationship with food intake and appetite is mainly studied with the action of 2-AG through CB1R, which is also involved in emotion, cognition, energy balance, pain, and neuroinflammation by regulating neurotransmitter release in the central nervous system ^[4]^. Kirkham et al reported that 2-AG injection into the nucleus accumbens shell, a brain region linked to eating motivation, potently and dose-dependently stimulated feeding, which was blocked by a CB1R antagonist ^[91]^. The levels of 2-AG in the hypothalamus declined during feeding, suggesting that fasting stimulates 2-AG, which acts through CB1R to induce food intake. Also, activation of cannabinoid CB1R in the parabrachial nucleus of the brain selectively stimulates the consumption of palatable foods high in fat and/or sugar, but not standard chow ^[92]^.

MAGL also contributes to the regulation of feeding behavior ^[56]^. In mice lacking MAGL, the preference for a high-fat diet was reduced relative to standard chow. When lipids were given orally, appetite was markedly decreased in MAGL-deficient mice and in CB1R/MAGL double knockout mice (DKO), whereas intraperitoneal lipid delivery did not produce the same effect. This depended on an intact vagus nerve, supporting a role for MAGL in gut-brain signaling and appetite regulation. Inhibition of MAGL, which degrades 2-AG, has been shown to protect against oxidative stress and mitochondrial dysfunction in the CNS, suggesting a role in maintaining neuronal metabolic homeostasis ^[93]^. MAGs contribute to the regulation of glucose homeostasis, thermogenesis, and insulin sensitivity, linking MAG metabolism to broader metabolic control mechanisms.

### 4.3 Effects on obesity, diabetes, liver, and cardiovascular disease

Roles of MAGs have also been implicated in metabolic disorders such as obesity, diabetes, and cardiovascular diseases. As closely linked with endocannabinoid signaling, several studies investigated 2-AG quantification in circulation as well as adipose tissue as a putative biomarker implication. For example, 2-AG was reduced in the subcutaneous adipose tissue (gluteal) of obese individuals and increased in both subcutaneous (visceral and gluteal) of the subjects following weight loss ^[94]^. However, tissue-specific expression levels and signaling via CB1R and CB2R may lead to different interpretations or consequences.

In adipose tissue, CB1R expression is higher than CB2R in adipocytes. Depot-specific CB1R expression in individuals with obesity was higher in visceral and subcutaneous abdominal adipose tissue and lower in gluteal fat ^[95]^. Paszkiewicz et al also demonstrated that in vitro CB1R expression levels are associated with adipogenic development/differentiation in 3T3-L1 adipocytes by using a CB1R antagonist, which led to elevated lipolysis and oxygen consumption rate ^[96]^. Additionally, CB1R agonist HU210 increased the accumulation of intracellular lipid droplets in mouse 3T3-F442A adipocytes in this study ^[97]^. Moreover, CB1R activation induces lipoprotein lipase activity in mouse primary adipocytes, which was blocked by preincubation with the CB1R selective antagonist SR141716A ^[98]^. Furthermore, deletion of CB1R leads to leanness, resistance to diet-induced obesity, and enhanced leptin sensitivity without changes in energy intake ^[99]^. Consistent with recovered sensitivity to endogenous leptin by treating peripheral CB1R antagonist JD5037 in DIO mice ^[100]^. These data support that CB1R signaling promotes fat synthesis and storage in adipocytes.

On the other hand, CB2R is known to exert both pro- and anti-inflammatory actions. CB2R agonists exacerbated obesity-associated inflammation, insulin resistance, and hepatic steatosis, as shown by increased inflammatory markers, such as CCL2 and TNFα ^[74]^. It aligns with Agudo et al that ablation of CB2R resulted in improved insulin sensitivity in obese, aging mice ^[101]^. Hosoki et al also reported that 2-AG enhances high-fat diet-evoked peripheral neuroinflammation, suggesting pro-inflammatory actions of 2-AG ^[102]^. Conversely, other researchers found that CB2R agonists reduced body weight, food intake, and fat mass in diet-induced obese mice ^[75]^. Conversely, CB2R deficiency in aged mice leads to obesity, inflammation, and hypertension ^[103]^. Rossi et al demonstrated that blocking CB2R using the AM630 reverse agonist increased inflammatory adipokine release and fat storage in white adipose tissue, suggesting CB2R as a potential anti-obesity target ^[104,105]^. It is in accordance with Wu et al reporting that the treatment of CB2R agonist JWH-133 reduced chronic inflammation and M1 macrophage activation in diet-induced obese mice via suppressing the Nrf2/HO-1 pathway ^[67]^. Moreover, when both CB1R/CB2R were ablated, mice were protected from diet-induced obesity, whereas CB2R^−/−^ displayed signs of impaired glucose clearance ^[106]^. It suggests that CB1R plays a prominent role in obesity, but the conflicting findings highlight the complex role of CB2R in obesity and metabolism and need further studies.

In contrast to pharmacologic manipulation, genetic ablation of MAGL showed a leaner phenotype and improved serum metabolic profile along with increased MAG content in the brain, liver, perigonadal white adipose tissue, and small intestine ^[55]^. MAGL KO mice had markedly reduced intestinal TAG secretion following an oral fat challenge, suggesting delayed lipid absorption. In line with this, Yoshida et al demonstrated that MAGL deficiency in mice ameliorated diet-induced obesity, improved insulin sensitivity, and altered fat absorption, with indicating its role in metabolic regulation independent of CB1R ^[56]^. This study also shows that intestinal 2-MAGs contribute to the regulation of food intake via gut-brain signaling mechanisms. In another study using the MAGL KO mouse model by Taschler et al, glucose tolerance and insulin sensitivity levels were improved in the absence of MAGL, and it was along with accumulated 2-AG in the brain and liver ^[107]^. Additionally, Poursharifi et al demonstrated the impact on the suppression of adipocyte-specific ABHD6, an isoform of MAGL, in which adipocytes present anti-inflammatory properties, along with increased MAG levels in the conditioned medium of adipocytes ^[108]^. Together, these suggest that MAGL deficiency attenuated diet-induced obesity and improved insulin sensitivity.

Beyond its roles as a signaling molecule, MAG serves as the obligate substrate for MGAT2-catalyzed DAG and subsequently TAG synthesis, and dysregulation of this esterification pathway is mechanistically linked to progressive liver disease. Excessive intestinal MGAT2 activity promotes dietary fat re-esterification and chylomicron assembly, increasing hepatic TAG delivery and contributing to the development of metabolic dysfunction-associated steatotic liver disease (MASLD) and its inflammatory progression to metabolic-associated steatohepatitis (MASH) ^[109]^. Consistently, *Mogat2*^*−*^/^*−*^ mice are protected from diet-induced hepatic steatosis, and it was further confirmed that intestinal MGAT2-mediated TAG synthesis is a key driver of hepatic lipid accumulation ^[51,110]^. Moreover, hepatic MGAT2 expression has been reported to be elevated in nonalcoholic fatty liver disease, whereas it is reduced after gastric bypass surgery, supporting that MGAT2 may contribute to MASLD ^[111,112]^. The potential for chronic hepatic lipid accumulation to progress from MASLD to MASH and, in some cases, to hepatocellular carcinoma indicates the long-term pathological significance of MAG-to-TAG flux dysregulation and suggests that MGAT2 is a promising therapeutic target in obesity-associated liver disease ^[113]^.

In addition, obesity is closely linked to type 2 diabetes/insulin resistance and pancreatic beta cell dysfunction. Matias et al, reported that 2-AG levels are significantly elevated in visceral adipose tissue and serum in type II diabetic patients, with no changes in subcutaneous adipose tissue ^[97]^. Also, using/in rat insulinoma RIN-m5F β-cells, high glucose treatment induced CB1R activation with a concomitant increase in 2-AG levels, resulting in insulin secretion. It indicates that CB1R signaling amplifies insulin secretion but may also contribute to β-cell stress under chronic hyperglycemia. Another study using isolated rat pancreatic β-cells and the clonal pancreatic β-cell line INS-1 832/13 demonstrated that treatment of 2-OG (25–400 µM) stimulated basal insulin secretion, and it was concomitant with increased ROS production. This stimulating effect of MAG on insulin secretion was reversed by inhibiting the activity of MAGL ^[114,115]^. In accordance with this prior finding, Zhao et al investigated the mechanism of MAG-mediated insulin secretion in β cells. They found that glucose-induced MAG production leads to insulin secretion, and this action is controlled by membrane-bound ABHD6 in β cells ^[116]^. Also, ABHD6 expression in β cells is inversely proportional to glucose-stimulated insulin secretion (GSIS), implying the suppressive role of ABHD6 in GSIS. They demonstrated that saturated MAG binds and activates the vesicle priming protein (exocytotic effector) Munc13-1, thereby induces insulin exocytosis, indicating MAG as a signaling molecule mediating GSIS and ABHD6 as a negative modulator of insulin secretion.

Lastly, cardiovascular disease is a major comorbidity in obesity. MAG and cardiovascular health are linked with cannabinoid receptor-mediated pathways. In a mouse model of atherosclerosis, ApoE/MAGL DKO exhibited increased plaque formation in the aorta but less vulnerable atherosclerosis, as shown by reduced lipids and macrophages, as well as elevated intraplaque smooth muscle staining and thicker fibrous caps ^[117]^. Treatment of CB2R inverse agonist during the last 3 weeks of 8-week Western diet feeding reversed the plaque phenotype in DKO mice, supporting pharmacological MAGL inhibition may reduce plaque rupture. It is in line with another mouse model of atherosclerosis-induced hyperlipidemia, in which both low-density lipoprotein receptor (Ldlr) and CB2R were deleted ^[118]^. CB2R deficiency in Ldlr-null mice aggravated atherogenesis by increasing lesional macrophage and smooth muscle cell content, reducing lesional apoptosis and altering extracellular matrix components, in part, by upregulating MMP9. On the other hand, a recent study by Avraamidou et al investigated the cell-specific modulation of CB2R in atherosclerosis by delineating its role using two cell-specific CB2R knockout mouse models in either myeloid or endothelial cells ^[119]^. Here, 2-AG exhibited an atherogenic effect, and it was abrogated in mice lacking myeloid expression of the CB2R, but not in mice lacking endothelial expression of the CB2R. These suggest that atherogenic 2-AG can be abrogated by inhibiting myeloid CB2R in mice. Overall, 2-AG modulates cell-specific plaque formation via CB2R in an atherosclerosis model, and the cell-specific action of 2-AG would be suggested to take into consideration for pharmacological treatment.

Several studies have shown that CB1R contributes to cardiovascular disease, despite mixed or opposing results compared to the CB2R-mediated effect. For example, short-term CB1R activation can protect against acute heart failure and pulmonary edema, as CB1R knockout mice showed higher mortality rates ^[120]^. However, chronic CB1R activation has shown detrimental effects by promoting oxidative stress, cell death, and cardiac dysfunction in models of acute and chronic cardiomyopathy in human cardiomyocytes ^[121]^, in which both studies demonstrate the significance of endocannabinoid homeostasis. Similarly, Rajesh et al explored that CB1R activation promotes JNK-MAPK signaling, inflammation, receptor for advanced glycation end products, ROS production, and fibrosis, which was suppressed by pharmacological inhibition of CB1R in a type 1 diabetic cardiomyopathy mouse model ^[122]^. Han et al addressed that CB1R and CB2R mediate differential effects on ROS production, as cannabinoid-induced ROS production by macrophages was CB1R-dependent, not CB2R, along with enhanced p38 MAPK-dependent monocyte chemoattractant protein 1 and TNF-α synthesis ^[123]^. It was also noted that CB1R protein was colocalized in CD68+ and CD36+ macrophages in human atheroma, implying that CB1R in combination with CD68+ and CD36+ contributes to atherosclerosis.

Although CB1R antagonists were addressed as potential therapeutic candidates, concerns about psychiatric side effects with systemic administration have limited their use ^[124]^. While CB1R overactivation may contribute to cardiovascular risk factors in obesity and metabolic syndrome, CB2R activation in immune and endothelial cells appears to limit inflammatory responses (immunomodulation) in atherosclerosis and reperfusion injury ^[125]^. Additional research would clarify whether MAG promotes atherosclerosis via CB2R and its cell-specific mechanisms.

### 4.4 MAG and inflammation/immunity

MAGs exhibit both pro-inflammatory and anti-inflammatory actions in a tissue/cell-specific manner. In response to LPS stimulation, 2-AG production was increased in both mouse J774 macrophages and circulating macrophages in rats ^[126]^, implying its role against inflammation/cellular stress. The authors also demonstrated that extracellular [^3^H]2-AG was cleared from the medium of intact J774 macrophages, as some stayed intact, while others esterified to phospholipids, DAG, and TAG, or hydrolyzed to [^3^H]arachidonic acid and glycerol. This indicates that 2-AG is taken up by macrophages, catabolized through several parallel enzymatic reactions in lipid metabolism, and participates in immunomodulation in macrophages. Moreover, 2-AG levels were elevated in response to oxidative stress in macrophages in vitro in a NADPH oxidase-dependent manner ^[127]^, indicating a positive correlation between heightened oxygen radical flux and 2-AG biosynthesis in macrophage cell lines and primary macrophages. It might be due to the antioxidant and/or anti-inflammatory effects of 2-AG, and oxyradical stress may be counteracted by the enhanced endocannabinoid tone.

MAGs could also play a distinct role in local macrophages. ATM are also associated with obesity, as characterized by crown-like structures. Miranda et al showed that blocking CB1R using AM251 attenuates obesity and adipose tissue inflammation via miRNA-mediated downregulation of Delta-like-4 in ATM ^[128]^, addressing a link between CB1R and ATM and the potential action of MAGs via CB1R. The significance of MAGs in metabolic regulation of ATMs was strengthened in a study by Poursharifi et al ^[5]^. Authors demonstrated that MAGs promote anti-inflammatory polarization of ATMs in a mouse model with genetic ablation of ABHD6 via 2-MAG/peroxisome proliferator-activated receptor (PPAR) signaling in obese mice. Treatment of metabolically activated RAW264.7 macrophages with ABHD6 inhibitor KT203 elevated 2-MAGs, including 2-PG (16:0), 2-stearoylglycerol (2-SG; 18:0), and 2-OG (18:1), while 1-MAG levels did not change, and it was associated with increased PPARα and PPARγ expression, further supporting MAG promotes anti-inflammatory response in mouse ATMs. Definitive evidence of MAGs in human adipose tissue or human ATMs is lacking.

In the liver, conflicting results are presented in direct/indirect relevance to MAG. CB2R agonist JWH-133 treatment protected mice from liver damage, as liver infiltration of mononuclear cells and pro-inflammatory cytokines were reduced ^[129]^. In addition, JWH-133 pretreatment of M1/M2-polarized macrophages significantly increased the secretion of anti-inflammatory cytokine IL-10 in M1 macrophages and potentiated the expression of M2 markers in M2-polarized macrophages ^[130]^, indicating a protective role of CB2R-mediated in liver injury. However, Habib et al demonstrated that MAGL inhibition reduces liver inflammation and fibrosis via modulating autophagy, but independent of CB2R activation or ABHD6 ^[131]^. Allaire et al showed that both hepatocyte- or myeloid-specific MAGL deletion delayed liver regeneration ^[132]^. The absence of MAGL in macrophages resulted in reprogramming macrophages towards a type I interferon pathway, which suppresses proliferation, and hepatocyte-specific MAGL deletion reduced liver eicosanoid production, in particular prostaglandin E2, which negatively impacts on hepatocyte proliferation. It was confirmed that human hepatocyte cell lines exposed to MAGL inhibitor MJN110 showed reduced hepatocyte proliferation, indicating MAGL promotes liver regeneration, mediating an inflammatory response. Similarly, 2-OG promoted liver inflammation and hepatic fibrosis in a mouse model of induced nonalcoholic steatohepatitis via activating GPR119-TGF-β1 signaling and stimulating ECM production in adjacent hepatic stellate cells ^[133]^. Authors addressed that the impact of different bioactive lipids on the inflammatory response is associated with specific cell types and environments, and macrophage-HSC crosstalk mediates NASH in mice fed a low choline + high fat-high sucrose diet, as 2-OG is a metabolic regulator/bioactive lipid.

Although indirect, 2-AG can also elicit an impact on inflammation. Rockwell et al demonstrated that IL-2 suppression by 2-AG was mediated by PPARγ in T cells independent of CB1R/CB2R ^[134]^. When 2-AG is oxidized by COX-2 and converted to prostaglandin H_2_ glycerol ester (PGH_2_-G), PGH_2_-G is subsequently metabolized by prostaglandin synthases to yield glyceryl prostaglandin species ^[22]^. Prostaglandin D synthase preferentially converts PGH_2_-G to prostaglandin D_2_ glycerol ester (PGD_2_-G), which exerts anti-inflammatory effects through activation of the PGD_2_ receptor 1 (DP1) receptor and PPARγ ^[23,135,136]^. The latter is proposed to occur via spontaneous dehydration of PGD_2_-G to 15d-PGJ_2_-G, a structural analog of the canonical endogenous PPARγ agonist ^[20,23]^. COX-2-dependent PG-G biosynthesis in macrophages occurs predominantly in the late phase of inflammatory activation, temporally dissociated from the early-phase arachidonic acid-driven prostaglandin response, and is dependent on DAG lipase-mediated 2-AG availability ^[137]^. Lastly, 2-AG can activate PPARα and PPARγ through both cannabinoid receptor-dependent and/or receptor-independent mechanisms, thereby mediating anti-inflammatory effects that extend beyond classical cannabinoid signaling ^[138]^. Together, these findings indicate that COX-2-mediated oxygenation of 2-AG generates bioactive lipid mediators with cell type-specific and context-dependent functions, and that the endocannabinoid and prostanoid systems intersect through this pathway to contribute to the resolution of inflammation.

## 5. Pharmacological approaches in MAG metabolism

**Figure 3.**
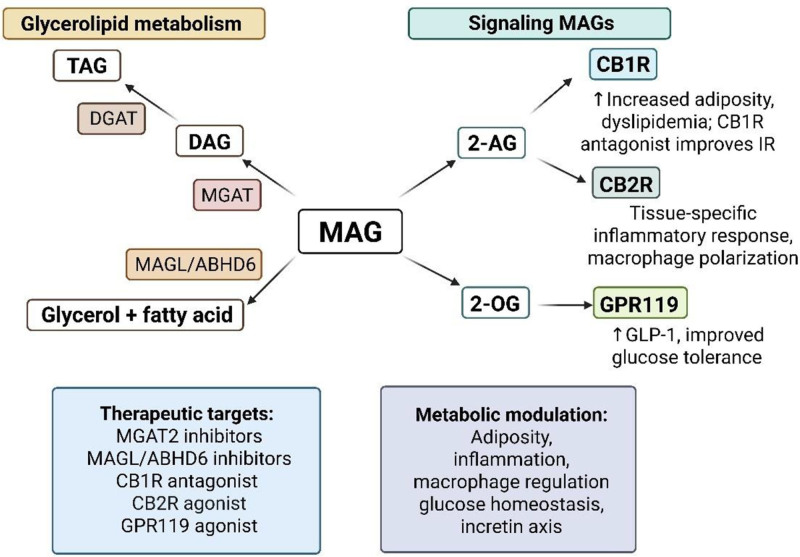
**Dual roles of MAGs at the crossroads of glycerolipid metabolism and signaling molecules.** (Left) MAGs can be re-esterified into DAG and subsequently into TAG for energy storage. This process is catalyzed by MGAT and DGAT, and inhibitors of these enzymes have been shown to reduce TAG accumulation, offering potential therapeutic strategies for metabolic disorders ^[139,140]^. Alternatively, MAG can be hydrolyzed into glycerol and FA by MAGL. Notably, LEI-515, a reversible and peripherally selective MAGL inhibitor, has demonstrated anti-inflammatory effects and the ability to alleviate oxidative stress in the liver, thereby improving metabolic outcomes in preclinical models ^[141]^. (Right) Two MAGs have been identified as important signaling molecules. First, 2-oleoylglycerol (2-OG) acts as a ligand for GPR119, which stimulates the secretion of incretins such as GLP-1 in the intestine, thereby enhancing insulin secretion and glucose homeostasis, similar to GPR119 agonists ^[142,143]^. Second, 2-AG is an endocannabinoid that activates CB1R and CB2R. Activation of CB1R promotes adiposity and metabolic dysregulation; thus, selective CB1R inverse agonists, such as JD5037, have been shown to improve insulin sensitivity and reduce body weight gain in preclinical models ^[100,144,145]^. In contrast, activation of CB2R appears to exert anti-inflammatory effects. For instance, the CB2R agonist JWH-015 has been reported to decrease body weight ^[75]^, and JWH-133 alleviates chronic inflammation in diet-induced obesity mouse models ^[67]^. However, further investigations are needed to fully elucidate the therapeutic potential and safety profile of CB2R-targeted interventions in metabolic diseases. 2-AG, 2-arachidonoylglycerol; 2-OG, 2-oleoylglycerol; DAG, diacylglycerol; DGAT, diacylglycerol acyltransferase; FA, fatty acid; GLP-1, glucagon-like peptide-1; GPR119, G protein-coupled receptor 119; MAG, monoacylglycerol; MAGL, monoacylglycerol lipase; MGAT, monoacylglycerol acyltransferase; TAG, triacylglycerol.

Current pharmacological approaches in drug development for modulating MAG metabolism are summarized in Figure 3.

### 5.1 MAGL inhibitor

MAGL inhibitors have been developed for therapeutic applications in inflammation, neurodegenerative disease, and cancer, including carbamates, disulfides, ureas, and tetrahydrolipstatin (THL)-based inhibitors. These compounds act on MAGL’s property as a serine hydrolase and target active cysteine residues ^[146]^. Several reviews suggest the potential aspects of MAGL inhibitors as drug targets for obesity, and various strategies led to application and development; however, targeting peripheral MAGL inhibitor development is ongoing to minimize adverse effects on the CNS. MAGL inhibitors can be categorized into two classes: irreversible and reversible. Although a covalent, irreversible inhibitor JZL184 was effective in alleviating inflammation, pain ^[147,148]^, it had major adverse effects on the CNS, and chronic administration of irreversible inhibitors as they have been shown to elicit CB1R downregulation and physical dependence over time ^[149]^. This finding led scientists to divert their attention to the development of more reversible inhibitors acting in peripheral tissues. Authors discovered an orally bioavailable reversible MAGL inhibitor, LEI-515, which increases 2-AG peripherally, not in the brain, using high-throughput screening and a medicinal chemistry program ^[150]^. LEI-515 attenuated liver necrosis, oxidative stress, and inflammation in a CCl4-induced acute liver injury model, without eliciting negative side effects in the CNS ^[141]^. Further preclinical work is needed to fully characterize the potential of MAGL modulation in obesity-related outcomes, as the endocannabinoid system plays a crucial role in metabolic homeostasis, and alternative strategies that exploit the beneficial therapeutic effects of MAGL inhibition without inducing deleterious consequences are highly desirable.

### 5.2 MGAT inhibitor

Recent progress in the phenotypic characterization of mice deficient in MGAT or DGAT has revealed important roles of these enzymes in the regulation of energy homeostasis and insulin signaling ^[151–153]^. Among MGAT isoforms, MGAT2 has garnered particular interest due to its predominant intestinal expression and its established role as the primary route for dietary fat re-esterification and absorption, and energy homeostasis (discussed in Section “Energy metabolism”). Selective inhibition of MGAT2 may thus provide novel treatment for obesity, diabetes, cardiovascular disorders, MASLD, and their related metabolic complications with improved efficacy and tolerability compared to other lipid-lowering approaches ^[109,154,155]^. Several MGAT2 inhibitors have been developed and shown efficacy across preclinical and clinical settings. Barlind et al identified and optimized novel MGAT2 inhibitors, demonstrating significant reductions in plasma TAG concentrations in animal studies ^[156]^. A novel oral MGAT2 inhibitor, S-309309 suppressed body weight gain and enhanced beta-oxidation in a high-fat diet mouse model ^[157]^, and subsequently demonstrated safety and tolerability in a phase I clinical trial ^[158]^.

MGAT2 inhibitor in HepG2 cells showed more effectiveness in lowering lipid accumulation ^[159]^, and pharmacological MGAT2 inhibition with Compound A improved hyperlipidemia, obesity, and diabetes in mice ^[139]^. The therapeutic relevance of blocking this pathway is further supported by the MGAT2 inhibitor BMS-963272, which reduced liver fibrosis and inflammation in murine NASH models and, in a Phase 1 clinical trial in adults with obesity, produced significant body weight reduction and elevated GLP-1 and PYY levels, providing translational evidence linking MGAT2 inhibition to both hepatic and systemic metabolic benefits ^[111]^. A patent review highlights the development of isozyme-selective inhibitors for MGAT and related acyltransferases, which may offer more targeted therapeutic options ^[160]^. These advances in MGAT inhibitor research show promise to treat metabolic syndrome and associated cardiovascular risks.

### 5.3 CB1R antagonist

Pharmacological administration for the modulation of CB1R or CB2R have been tested as a therapeutic approach for obesity. Cannabinoid CB1R antagonists have emerged as a promising therapeutic approach for obesity treatment ^[161]^. Rimonabant (SR141716A), the first CB1R selective antagonist approved and used in clinical practice, has shown to significantly reduce body weight and improve metabolic parameters such as insulin sensitivity, lipid profiles, and waist circumference in multiple clinical trials ^[162–164]^. However, psychiatric side effects led to its withdrawal from the market ^[165]^. Recent research focused on developing peripherally restricted CB1R antagonists to maintain efficacy while minimizing central nervous system-related adverse effects ^[166]^. The development of CB1R antagonists has been largely based on modifications to rimonabant’s 1,5-diarylpyrazole structure ^[164]^; however, additional strategies have been explored, including increasing polar surface area, decreasing lipophilicity, and designing neutral antagonists and allosteric inhibitors ^[167]^. Despite challenges, CB1R antagonists remain a promising avenue for obesity treatment, with ongoing efforts to optimize their safety and efficacy profiles ^[168]^. Another selective, peripheral CB1R antagonist, TXX-522 ameliorated insulin resistance in high-fat diet-induced obese mice ^[144]^. This group confirmed that there was no impact on food intake in this model, validating the limited brain penetration of this compound with the potential to control obesity and related metabolic disorders. Also, the CB1R inverse agonist JD5037 has been reported to ameliorate diet-induced obesity, improving metabolic parameters without central side effects, suggesting its potential to reduce cardiometabolic risk ^[169]^. Currently, a monoclonal antibody targeting peripheral CB1R is undertaking Phase 2 trials (Skye Bioscience), showing post-treatment maintenance of weight loss, and a second-generation inverse agonist CRB-913 is in a Phase 1 trial (Corbus Pharma) with enhanced lipolysis and energy metabolism resulting in a reduction in appetite and body weight. These candidate drugs may be potent anti-obesity medicines as standalone and/or in combination with FDA-approved incretin mimetics (eg, semaglutide, tirzepatide).

### 5.4 CB2R agonist/antagonist

CB2R agonist JWH-133 has been shown to reduce inflammation and characteristics of obesity in human primary subcutaneous adipocytes ^[104]^. This effect was confirmed by CB2R blockade with AM630 where inflammatory adipokine release and fat storage were increased and reduced browning. Similarly, injection of CB2R agonist JWH-133 decreased body weight, food intake, adipocyte size, and fat mass in high-fat diet-fed mice ^[75]^. It was associated with increased fasting plasma non-esterified fatty acids and decreased fasting insulin, TAG, indicating CB2R activation increases lipolysis and an anxiolytic response. In addition, CB2R agonist JWH-133 at 5 to 10 mg/kg BW reduced body weight gain and M1-associated cytokine production and improved insulin sensitivity in a diet-induced obesity mouse model via the Nrf2/HO-1 pathway ^[71]^, suggesting CB2R activation potentiates a protective role in obesity and metabolic dysregulation. However, another study assessed that both CB2R agonist AM1241 and antagonist AM630 have shown reduced circulating leptin levels without weight loss, similarly by pharmacological administration in a diet-induced mouse obesity model ^[170]^. Together, CB2R -mediated signaling contributes to inflammation and obesity, but further investigations would support delineating specific pathways of action.

### 5.5 GPR119 agonist

GPR119, a GPCR expressed in pancreatic β-cells and gastrointestinal enteroendocrine cells, has emerged as a promising target for treating type 2 diabetes and obesity ^[80,81]^. 2-OG has been identified as a ligand/agonist for GPR119, and its role in promoting incretin production has been described ^[69]^. Orally available GPR119 agonists have demonstrated the ability to lower blood glucose without causing hypoglycemia, slow diabetes progression, and reduce food intake and body weight in animal models ^[171]^. These agonists offer a unique dual mechanism of action, elevating both insulin and incretin levels in a nutrient-dependent manner ^[172,173]^. Along with several GPR119 agonists entering clinical trials and showing promising results in glycemic control and incretin release, MAG holds potential for improving type 2 diabetes treatment ^[174]^.

## 6. Considerations and limitations in MAG research

2-AG plays crucial roles both as an intermediate in lipid metabolism and as a signaling molecule ^[175]^. Thus, measurement of 2-AG from tissue samples represents both “signaling” and “metabolic-intermediate” levels of 2-AG, and it is likely that only a small fraction of the 2-AG measured in tissue samples is functioning as an endocannabinoid ^[176]^. Additionally, most current studies measure MAGs by extracting whole tissues and running liquid chromatography-mass spectrometry (LC-MS) on the homogenate at one time point ^[177,178]^. This challenges the interpretation of whether measured MAGs come from rapidly changing signaling pools or from larger, slow turnover pools used for general lipid storage metabolism. Alternative/accurate options for specific cell population/cell-specific lipidomics include isolated/sorted cells using fluorescence-activated cell sorting in several studies or chemical conjugation strategies for single-cell MS analysis; however, MAGs, relatively at low abundance, remain to be further expanded and tailored for improved detection at higher resolution ^[179]^.

## 7. Conclusions

This review highlighted the complex interplay between MAGs and various physiological processes across different tissues. MAGs serve as important intermediates in both anabolic and catabolic pathways of lipid metabolism, influencing energy storage and utilization. MAGs, particularly 2-AG and 2-OG, also function as signaling molecules through interactions with cannabinoid receptors (CB1R and CB2R) as well as GPR119, respectively, influencing inflammation, cardiovascular function, and insulin secretion. Tissue-specific actions of MAGs in the intestinal epithelium, adipose tissue, liver, and central nervous system contribute to the pathophysiology of obesity and type 2 diabetes, and liver disease. Lastly, reviewing therapeutic targets of MAG metabolism, including MGAT, MAGL, cannabinoid receptor, and GPR119, provides insights into pivotal aspects of MAG for further research.

## Author contributions

M.Y.P., J.B., and J.O.A. designed the overall outline of the mini-review; M.Y.P. and A.M. conducted the literature search, search analyzed published data, and wrote the manuscript. All authors have read, edited, and approved for the final manuscript.

## Conflicts of interest

The authors declare no conflict of interest.

## Funding

This work was supported by NIH grants (K08-DK117064 and P01-HL160470).
